# Using quantitative and analytic EEG methods in the understanding of connectivity in autism spectrum disorders: a theory of mixed over- and under-connectivity

**DOI:** 10.3389/fnhum.2014.00045

**Published:** 2014-02-26

**Authors:** Robert Coben, Iman Mohammad-Rezazadeh, Rex L. Cannon

**Affiliations:** ^1^Neurorehabilitation and Neuropsychological ServicesMassapequa Park, NY, USA; ^2^Integrated Neuroscience ServicesFayetteville, AR, USA; ^3^Center for Mind and Brain, University of CaliforniaDavis, CA, USA; ^4^Semel Institute for Neuroscience and Human Behavior, University of CaliforniaLos Angeles, CA, USA; ^5^Psychoeducational NetworkKnoxville, TN, USA

**Keywords:** autism spectrum disorders, EEG/MEG, connectivity analysis, coherence analysis, sLORETA, granger causation analysis

## Abstract

Neuroimaging technologies and research has shown that autism is largely a disorder of neuronal connectivity. While advanced work is being done with fMRI, MRI-DTI, SPECT and other forms of structural and functional connectivity analyses, the use of EEG for these purposes is of additional great utility. Cantor et al. (1986) were the first to examine the utility of pairwise coherence measures for depicting connectivity impairments in autism. Since that time research has shown a combination of mixed over and under-connectivity that is at the heart of the primary symptoms of this multifaceted disorder. Nevertheless, there is reason to believe that these simplistic pairwise measurements under represent the true and quite complicated picture of connectivity anomalies in these persons. We have presented three different forms of multivariate connectivity analysis with increasing levels of sophistication (including one based on principle components analysis, sLORETA source coherence, and Granger causality) to present a hypothesis that more advanced statistical approaches to EEG coherence analysis may provide more detailed and accurate information than pairwise measurements. A single case study is examined with findings from MR-DTI, pairwise and coherence and these three forms of multivariate coherence analysis. In this case pairwise coherences did not resemble structural connectivity, whereas multivariate measures did. The possible advantages and disadvantages of different techniques are discussed. Future work in this area will be important to determine the validity and utility of these techniques.

## Introduction

Autistic Spectrum Disorders (ASD) are a heterogeneous group of pervasive developmental disorders including Autistic Disorder, Childhood Disintegrative Disorder, Pervasive Developmental Disorder-Not Otherwise Specified (PDD-NOS), and Asperger Disorder. Children with ASD demonstrate impairment in social interaction, verbal and nonverbal communication, and behaviors or interests (DSM-IV-TR; APA, 2000). ASD may be comorbid with sensory integration difficulties, mental retardation or seizure disorders. Children with ASD may have severe sensitivity to sounds, textures, tastes, and smells. Cognitive deficits are often associated with impaired communication skills. Repetitive stereotyped behaviors, perseveration, and obsessionality, common in ASD, are associated with executive deficits. Executive dysfunction in inhibitory control and set shifting have been attributed to ASD (Schmitz et al., 2006). Seizure disorders may occur in one out of four children with ASD; frequently beginning in early childhood or adolescence (NIMH, 2006).

Research reviewing the epidemiology of autism (Center for Disease Control and Prevention; CDC, 2009) reported between 1 in 80 and 1 in 240 children in the United States diagnosed with the disorder. A report of just 3 years ago (CDC, 2009) suggested a prevalence of 1 in 110, and as high as 1 in 70 boys. In their most recent report, the CDC (2012) suggests that the rate has risen to 1 in 88. ASDs are five times more likely in boys for which it is seen in 1 out of 54 male children. According to Blaxill (2004), the rates of ASD were reported to be <3 per 10,000 children in the 1970s and rose to >30 per 10,000 in the 1990s. This rise in the rate of ASD constituted a 10-fold increase over a 20 year interval in the United States. These findings make accurate assessment of autistic individuals and their underlying neurophysiology a priority.

## EEG assessment in autism

Multiple neuroimaging studies have demonstrated brain anomalies in autistics compared to healthy controls (McAlonan et al., 2004; Page et al., 2006). The electroencephalogram (EEG) was one of the earliest techniques used to investigate the neurobiology of autism (Minshew, 1991). The recognition of a high instance of EEG abnormalities and of seizure disorders in the autistic population was among the earliest evidence of a biologic basis for the disorder (Minshew, 1991). Moreover, the EEG is a premiere tool to assess neural dysfunctions related to autism and seizures due to its' noninvasive nature, availability and utility in detailing these types of difficulties.

Recent analyses have estimated the prevalence of seizure disorders in autistic series at anywhere from 20 to 46%. Based on recent analyses, the prevalence of seizure disorders in autistic series is estimated at about 36% (Danielsson et al., 2005; Hughes and Melyn, 2005; Hara, 2007; Parmeggiani et al., 2007). In fact, it has been reported that the autistic population has about 3- to 22-fold increased risk of developing seizure disorders as compared to the normal population (Volkmar and Nelson, 1989). Sub-clinical seizure activity or paroxysmal discharges occur in an even higher proportion of autistics, but the significance of these remain uncertain (Hughes and Melyn, 2005; Parmeggiani et al., 2007). Ray et al. (2007) have suggested that the initial phase of cortical spikes may relate to underlying intracranial foci. Other work has suggested that EEG spikes may reflect underlying morphological brain abnormalities (Shelley et al., 2008) and/or metabolic disturbances (Kobayashi et al., 2006).

In a recent study, Parmeggiani et al. (2010) demonstrated that in a large inpatient sample 58% of adults with autism aged 20 or older had experienced epilepsy or a seizure during their lifetime. For these reasons, experts in the field have recommended the use of routine and sleep EEGs in the evaluation of autistic disorders, especially when there has been regression or there are signs of possible seizures. In fact, seizure detection has been the primary role of the EEG for decades. When EEG assessment is processed and analyzed with the most advanced techniques it can be invaluable for screening for possible seizures, evaluation of autistic disorders, and assessing the neurophysiological challenges of children with ASD. While brain structural imaging may reveal interesting findings, assessment of regional brain dysfunction is more revealing and usually requires functional brain imaging techniques. This would include techniques such as functional MRI, PET, single photon emission computed tomography, magnoencephalography (MEG), and even EEG. Some of these techniques require sedation or injection of radioactive material so as to make participation difficult for a typical autistic child. EEG, however, appears to be the most clinically available and again least invasive of these techniques. Further, it has been found that unique patterns of regional dysfunction could be discerned through the quantitative analysis of the EEG.

## Quantitative EEG findings and ASD

A review of the existing literature identified 14 studies that used quantitative techniques to analyze differences in EEG (QEEG) activity between children with ASD and normal controls with conflicting results. Two studies showed decreased delta frontally (Dawson et al., 1982; Coben et al., 2008), while one found increased activity in the delta frequency range (Murias et al., 2007). Two studies reported increased generalized delta or described “slowing” (Cantor et al., 1986; Stroganova et al., 2007). Two studies showed theta increases (Small et al., 1975; Coben et al., 2008), while one study reported reduced theta (Dawson et al., 1982). By contrast, findings have been quite consistent within the alpha through gamma frequency range. All studies reported reduced alpha power (Dawson et al., 1982; Cantor et al., 1986) and increased beta (Rossi et al., 1995; Chan and Leung, 2006; Coben et al., 2008) and gamma power (Orekhova et al., 2006). Multiple studies report a lack of hemispheric differences in QEEG spectral power in autistic samples compared to findings of hemispheric differences in normal controls. Autistic children showed decreased power asymmetry when compared to normal or mentally handicapped controls (Dawson et al., 1982; Ogawa et al., 1982). Three studies investigated cortical connectivity in ASD samples using QEEG coherence measures, with all reporting reduced connectivity, especially over longer distances (Cantor et al., 1986; Lazarev et al., 2004; Coben et al., 2008). One concern has been that sample sizes by and large have not been large enough to allow for investigation of the observed inconsistencies in findings reported above.

In the largest study of its' kind, we (Coben et al., 2013) included a total of 182 children, 91 on the autistic spectrum and 91 healthy controls. Findings indicated an absolute delta deficit over frontal and central brain regions and theta excesses over frontal, temporal and posterior regions for the ASD sample. There were significant relative theta excesses over frontal and temporal regions, alpha and beta excesses over multiple regions. Interestingly, cluster analytic techniques were used and able to delineate qeeg subtypes of ASD. Furthermore, a discriminant function analysis was able to correctly identify ASD children at a rate of 95%. Despite power subtypes having been shown, VARETA (di Michele et al., 2005) revealed similar sources of activation including temporal, posterior cortical and various limbic regions. These findings raise the likelihood that the study of neuronal networks in autism may lead to a greater understanding of ASD than localization of brain activity. Power asymmetry and coherence findings were also significant consistent with evidence supporting the notion of frontal hypercoherence and anterior to posterior temporal hypocoherences. These findings suggest that the brain dysfunction in autistic disorders is often bilateral and impacts both anterior and posterior axes. Alternatively, one could view the brain dysfunction in autism as an abnormality in connectivity that disrupts function in multiple regions (Minshew and Williams, 2007). This would suggest that such connectivity impairments are prevalent in autistic children. This is consistent with the findings of Coben et al. (2008). Such an interpretation is also supported by the literature suggesting that autism is primarily a disorder of neural connectivity.

## Autism as a disorder of neural connectivity

There is increasing evidence that the cardinal disruptions in autism are represented by disruptions in brain connectivity (Courchesne and Pierce, 2005; Minshew and Williams, 2007; Mak-Fan et al., 2012). There is mounting evidence of head enlargement as a result of brain overgrowth early in life (first 1–2 years) (Courchesne et al., 2001, 2003) as a result of enhancements in frontal white matter and minicolumn pathology (Casanova et al., 2002; Herbert et al., 2004; Carper and Courchesne, 2005; Vargas et al., 2005). This overgrowth, then, leads frontal over-connectivity (Courchesne and Pierce, 2005; Coben and Myers, 2008; Rinaldi et al., 2008) which interferes with the normal developmental trajectory. This disruption, theoretically, then halts the natural developmental progression in which anterior to posterior brain regions would enhance their synchronization and specialization of fucntions (Damasio, 1989; Supekar et al., 2009). This pattern, in fact, was observed in our data above showing frontal hypercoherence and bilateral temporal hypocoherences (Coben et al., 2013).

Other data support this hypothesis as well. For example, Mak-Fan et al. (2012) examined changes in diffusivity with age within frontal, long distant, longitudinal and interhemispheric tracts across ages 6–14. Their findings showed that while typically developing controls change and evolve on such measures children with autism did not. This suggests that such connectivity difficulty exist and persist in such children. More specifically, frontal and local (short neuronal paths) hyperconnectivity has been shown to be present in autistic samples (Wass, 2011; Li et al., 2014). In addition, there is other recent data showing hypoconnectivity in long distance and posterior to anterior or temporal regions in autistics. Isler et al. (2010) have shown low interhemispheric coherence in visual evoked potentials in such children. Studies of functional connectivity related to visuospatial processing and the social-emotional processing networks have also shown reduced connectivity compared to healthy controls (Ameis et al., 2011; McGrath et al., 2012; von dem Hagen et al., 2013). Similarly, low functional connectivity has been shown to relate to poor language processing in autistic children (Kana et al., 2006). Many of these studies used 3-dimensional imaging techniques such as MRI, fMRI or DTI (diffusion tensor imaging). Interestingly, EEG/QEEG studies of coherence have shown similar findings. Coben et al. (2013) have recently shown findings consistent with frontal hypercoherence and bilateral posterior-temporal hypocoherences. Similarly, high frontal coherence has been observed in other studies (Coben and Padolsky, 2007; Murias et al., 2007). In addition, EEG technology has been able to demonstrate long range, anterior to posterior and temporal hypocoherences (Murias et al., 2007; Coben et al., 2008). All of these coherence findings have been based on measurements between pairs of electrodes. There is reason to believe that more advanced statistical approaches to EEG coherence may provide more detailed and accurate information.

## Pairwise vs. multivariate coherence estimates

Traditionally and historically EEG coherence estimates have arisen from cross correlations between pairs of electrodes (Bendat and Piersol, 1980). Such a calculation is often performed within a given frequency range and is normalized for amplitude or magnitude. As such the following equation serves as the operational definition:

(1) $$\tau_{xy}^{2}(f)=\frac{{\left(G_{xy}(f)\right)}^{2}}{\left(G_{xx}(f)G_{yy}(f)\right)}$$

Where: ***G***_***xy***_(***f***) = cross power spectral density and

***G***_***xx***_(***f***) and ***G***_***yy***_(***f***) = auto power spectral densities

The final normalized coherence value is given by Equation (2):

(2) $$\tau_{xy}^{2}(f)=\frac{r_{xy}^{2}+q_{xy}^{2}}{G_{xx}G_{yy}}$$

Where: ***r***^2^_*xy*_ = real cospectrum and ***q***^2^_*xy*_ = imaginary quadspectra

***G***_***xx***_(***f***) and ***G***_***yy***_(***f***) = as in Equation (1)

Phase: ***159.1549 tan* − *1*(***q***/*r***)/***fc***

Where: ***r*** and ***q*** = as in Eq.2; ***fc*** = center frequency of filter

For a more detailed explanation or discussion of these please see Otnes and Enochson (1972) and Thatcher et al. (1986). These concepts have been used and applied commonly. In fact, a search in Google Scholar for “EEG coherence pairs” revealed more than 14,500 citations. While this approach has been commonly used in the past, there are certain limitations in its application and accuracy. First, there is a confound in pairwise coherence measurements, namely the notion of electrode distance. It has been observed that the further the distance between electrodes the lower their coherence value will be regardless of their functional connectivity, with distances as long as at least 5 cm. (Nunez, 1994; Nunez and Srivinasan, 2006; Thatcher et al., 2008). Pairwise coherence measures for nearby electrodes are biased by volume conduction, to a degree that varies as a function of inter-electrode distance such that physically closer pairs manifest higher coherence values. While statistical corrections have been offered for these concerns (Nunez et al., 1997; Barry et al., 2005), multivariate approaches that may eliminate this problem should be desired.

Other reasons for concern include a vast array of possible comparisons (171 comparisons in one frequency band), and that many of these pairs do not correspond to known neuronal pathways. Lastly, pairwise coherence estimates are not precise in their anatomical locations as there is a presumption of a two dimensional and not a 3-dimensional space (Black et al., 2008). It has further been observed that multivariate strategies to assess coherence metrics are more accurate and effective than their pairwise counterparts (Kus et al., 2004; Barry et al., 2005; Pollonini et al., 2010). For example, Duffy and Als (2012) used principal components analysis of coherences (multivariate approach) and demonstrated the ability to distinguish between children with autism and neurotypical controls.

## Multivariate approaches to coherence analysis

Multivariate, advanced statistics models, have rarely been applied to the issue of coherence in the autistic brain. With these new advances in analytic methods it is hoped that we will come closer to understanding these dynamic phenomena. Hudspeth (1994) was one of the first to investigate a multifactorial representation of EEG covariance. He and his students obtained multichannel EEG data and computed all combinations and similarities and differences among the waveforms to produce a triangular correlation matrix for each subject. The correlation matrices were then factored with principal components analysis to obtain three eigenvectors and the weighting coefficients required to project each of the waveforms into a 3-dimensional geometric representation of the cortical surface of the brain. When processed in this way, this integration of factored data reduces the redundancy in the EEG waveforms and patterns and correspond to known neural network pathways. This is the predecessor of Duffy and Als (2012) with enhanced complexity. The first three principle components are summed to create a 3-dimensional representation of these multivariate coherences. When EEG data is represented in this way, the resulting eigenimages reveal similarities and differences across systems in the brain often grouped together by cortical function or neuronal systems. Deviations from these expected relationships points to dysfunctional aspects of coherence. EEG data is gathered based on the classic 10–20 international system/electrode configuration (Jasper, 1958). In this system of analysis, these points in space are redrawn in 3-dimensional space based on each locations' multidimensional relationship with all other locations based on horizontal, sagittal and coronal views. As such, connectivity patterns are determined by the inter-relationships among all combinations of inputs and are thus considered multivariate or multi-source in nature.

**Figure 1:**
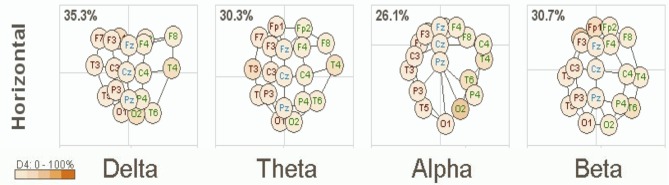
**NeuroRep Multivariate Connectivity analyses showing eigen images in the horizontal place across delta, theta, alpha, and beta frequencies**. Observable features include; (1) right hemisphere (temporal) hypocoherences across all frequency bands, (2) hypercoherences in the alpha band over prefrontal regions, and (3) right parietal-posterior temporal hypercohences in the theta and alpha frequency bands.

A clinical example of this is now presented below in Figure 1. This is based on an EEG recording performed with a 12 year old girl diagnosed with autism with her eyes open and fixed on a spot directly in front of her. Her most prominent clinical feature included very limited social skills. The EEG data was consistent with a mu rhythm (Kuhlman, 1978) that does not suppress to movement or observation of social scenes (Oberman et al., 2007) and is, thus, considered indicative of mirror neuron dysfunction (Oberman et al., 2005). This system of coherence assessment was created by Hudspeth (2006) and is contained within the NeuroRep QEEG Software system. The method of calculation has been described above as these eigen images can be viewed as an image in 3-dimensional space representing the functional proximity or coherence among the various electrodes based on the 10/20 International EEG recording system (Niedermeyer and Lopes da Silva, 2004). As such, electrode positions that are closer in proximity reflect greater hypercoherences and electrodes that are further apparent are indicative of greater hypocoherences. As may be seen in Figure 1 this analysis reveals a pattern of mixed hypo and hypercoherences with prefrontal and parietal-posterior temporal regions being hyperconnected among themselves and large regions of hypocoherences across much of the right hemisphere but especially from posterior frontal to posterior temporal regions.

## sLORETA functional connectivity

Standardized low-resolution brain electromagnetic tomography (sLORETA) is a method of probabilistic source estimation of EEG signals in standardized brain atlas space utilizing a restricted inverse solution (Pascual-Marqui et al., 1994, 2002). sLORETA has been used to examine EEG sources in depression (Pizzagalli et al., 2003), epilepsy (Zumsteg et al., 2006), and evaluating temporal changes associated with differential task specific default network activity (Cannon and Baldwin, 2012). Recently, sLORETA and fMRI were shown to localize DMN regions with complementary accuracy (Cannon et al., 2011). Recent statistical and theoretical advances have led to the use of this technology in the measurement of source coherences (Pascual-Marqui, 2007).

There has been rigorous discourse over the localization accuracy of low-resolution electromagnetic tomography (LORETA) and its evolution toward standardized low-resolution electromagnetic tomography (sLORETA) (Pascual-Marqui et al., 1994; Pascual-Marqui, 2002). The most important issue at hand for any EEG localization or functional neuroimaging technique is the fact that none of these methods localize the “true” source, rather they model the source with probabilistic techniques. This includes all methods that utilize statistical/mathematical modeling, including functional magnetic resonance imaging (fMRI) and magnetoencephalography (MEG) (Knyazev, 2013). Thus, when using sLORETA in this fashion, we do operate under certain assumptions/restrictions. First, we are restricted to cortical gray matter; including the hippocampus and the computations and source estimations are restricted by geometric constraints. Additionally, in the most basic sense it would be optimal to evaluate the source estimates provided by sLORETA to an individual's specific MRI scan, thus we utilize a standardized MRI from the Montreal Neurological Institute with 6340 5 mm^3^ voxels and with it the potential error (Collins et al., 1994). In the localization of EEG sources, recent works have shown the sLORETA and LORETA methods to improve and even outperform other methodologies in accuracy (Grech et al., 2008; SaeidiAsl and Ahmad, 2013) with the addition of regularization parameters. Additionally, standardized LORETA is not a modification of the original LORETA, rather it does not utilize the Laplacian operator, instead it utilizes standardized current density.

Importantly, for this particular single case study we extrapolated CSD for each frequency range to enter into bivariate procedures to compute the person correlation coefficient for the mean total relative current source density for each of the ROIs included in this study. For larger sample sizes, each frequency domain can be analyzed and the results do not correspond to issues with excessively high correlations in neuroimaging studies as reported in Vul et al. (2009), rather it appears that task and subjective mental activity are important to understanding functional coupling that occurs within and between networks in the human brain (Cannon and Baldwin, 2012). The basis for using a correlation procedure is that functional relationships between groups of neurons within the brain can exist, even if the structural relationships are unknown. We have evaluated the use of correlations using two neuroimaging methods (sLORETA/fMRI) with accurate results in the default network (Cannon et al., 2011, 2012). In any experiment utilizing discrete or distributed sources of the EEG volume conduction is a formidable concern. In short, volume conduction decreases as a function of distance from a current source at zero phase lag; however, if volume conduction is a problem in any sense then phase lag differences must be near zero and remain near zero independent of distance (Kauppinen et al., 1999; Thatcher et al., unpublished manuscript).

The distributed source localization problem and its solution as computed by sLORETA can be stated as (Pascual-Marqui, 2002; Liu et al., 2005)

(3) Φ=KJ+c1

Where Φ is an *N* × 1 vector containing the scalp electric potentials measured from N_*E*_ electrodes on the scalp, **J** is a 3*M* × 1 vector representing current sources at *M* locations within the brain volume, with three orthogonal components per location and *c* being a common reference. **K** is the lead filed matrix representing the system transfer coefficients from each source to each measuring point (Pascual-Marqui, 2002). Regularization using a zero-order Tikhonov-Philips cost function permits a unique solution to Equation (1) (Hansen, 1994)

(4) $$\underset{J}{\min}\left\{{\left\|\Phi -KJ\right\|}^{2}+\alpha {\left\|J\right\|}^{2}\right\}$$

Where α is the regularization parameter using the L-curve method. The source estimation is then derived as

(5) $$\hat{J}=T\Phi$$

where

(6) $$T=K^{T}{\left[KK^{T}+\alpha I\right]}^{-1}$$

Substituting (3) into (5) yields

(7) $$\hat{J}=TKJ=K^{T}{\left[KK^{T}+\alpha I\right]}^{-1}KJ=RJ$$

where **R** is the resolution matrix, defined as

(8) $$R=KT{\left[KKT+\alpha I\right]}^{-1}K$$

The resolution matrix illustrates a map from the authentic source activity to the estimated activity, with **R** being an identity matrix. Thus, the basic functional concept of sLORETA is to normalize the estimation using a block-by-block inverse of the resolution matrix using (8)

(9) $${\hat{J}}_{l}^{T}(R_{ll})-1{\hat{J}}_{l}$$

where **Ĵ**_*l*_ is a 3 × 1 vector of the source estimate at the lth voxel and **R**_*ll*_ is a 3 × 3 matrixcontaining the *lth* diagonal block of the resolution matrix. sLORETA was shown to give the best performance in terms of localization error and ghost sources, with different noise levels (Grech et al., 2008).

### Methods

**Figure 2:**
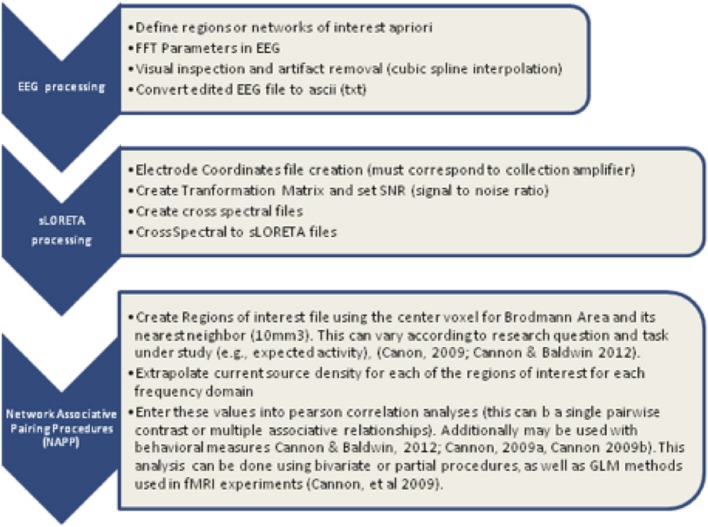
**Procedure to examine the associations between the center voxel within a specified Brodmann Area (BA) and its nearest neighbor (10 mm^3^)**. Listed in the figure from **top** to **bottom** are the steps used to process EEG data and create the correlation maps between regions of interest (ROIS). In short, EEG data must be processed first with careful attention given to artifact contamination and its potential influence across all steps of the sLORETA procedures. The next step is to create the sLORETA files in order to extract the CSD at specified ROIs. Finally, using any statistical program the correlations between the ROID, or networks of interest can be contrasted for functional associations.

A region of interest (ROI) file with the MNI coordinates for the 15 seed points for the center voxel within Brodmann Area (BA) regions was constructed (see Table 1). These ROIs were selected apriori based on their known involvement in the mirror neuron system and social perceptual networks. Each of the ROI values consisted of the mean current source density from each ROI seed and one single voxel (its nearest neighbor) for total voxel size 10 mm. The resulting file produced the average current source density for each frequency domain across multiple EEG segments for all subjects for each seed (ROI). The CSD data for each frequency band were organized into Microsoft Excel spreadsheets and then entered into SPSS 19 for analysis. sLORETA images corresponding to the estimated neuronal generators of brain activity within each given frequency range were calculated (Frei et al., 2001). This procedure resulted in one 3D sLORETA image for this single subject for each frequency range. We entered each frequency domain into the analysis for an N of 4 (delta 0.5–4.0 Hz; theta 4–8 Hz; alpha 8–12 Hz, and beta 12–32 Hz). The sequence of steps involved in generating the sLoreta source coherence image is presented in Figure 2.

**Table 1:** **ROIs for this study: in the table from left to right are the x, y, and z MNI coordinates for center voxel, Lobe, structural nomenclature and Brodmann Area**.

**X-MNI**	**Y-MNI**	**Z-MNI**	**Lobe**	**Structure**	**Brodmann area**
50	20	15	Frontal lobe	Inferior frontal gyrus	45
30	25	−15	Frontal lobe	Inferior frontal gyrus	47
45	35	20	Frontal lobe	Middle frontal gyrus	46
25	55	5	Frontal lobe	Superior frontal gyrus	10
20	45	−20	Frontal lobe	Superior frontal gyrus	11
40	−5	10	Sub-lobar	Insula	13
25	−75	10	Occipital lobe	Cuneus	30
45	−20	−30	Temporal lobe	Fusiform gyrus	20
5	−45	25	Limbic lobe	Posterior cingulate	23
0	20	20	Limbic lobe	Anterior cingulate	33
20	−10	−25	Limbic lobe	Parahippocampal gyrus	28
10	−50	35	Parietal lobe	Precuneus	31
5	30	20	Limbic lobe	Anterior cingulate	24
45	−55	−15	Temporal lobe	Fusiform gyrus	37
40	15	−30	Temporal lobe	Superior temporal gyrus	38

**Figure 3:**
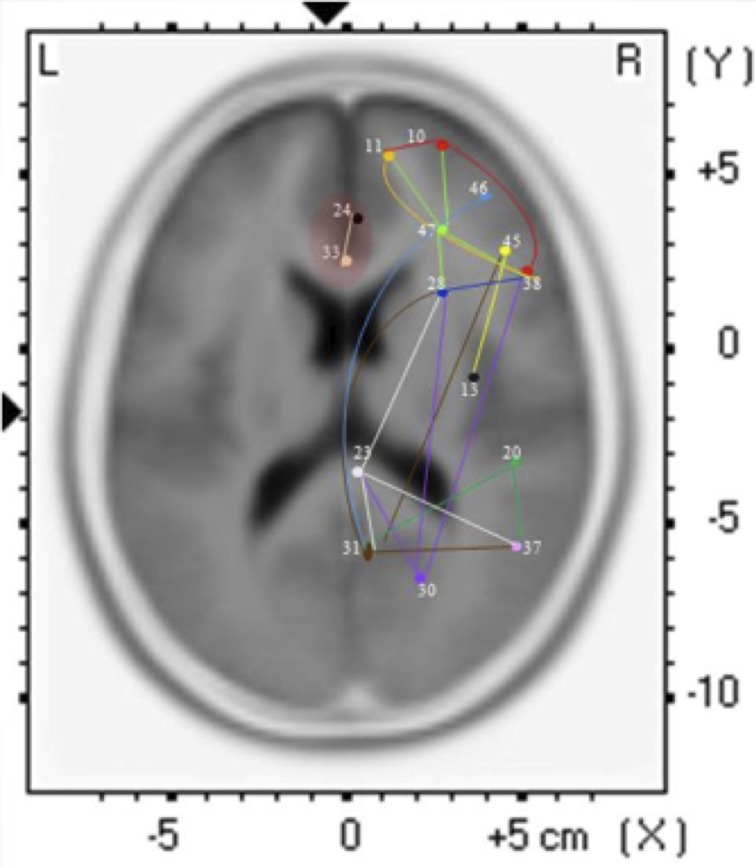
**Each of the 15 ROIs for this case study are represented in a different color**. The lines indicate significant correlations between the colored ROI and other regions. The color of the line is the same as the ROI in relation to its functional connectivity with other ROIs.

The findings for this same case as described above are presented in Figure 3. The most apparent findings from this analysis seem to be regions that are overconnected with each other and that these regions often involve close neighbors or regions of close proximity (see Table 2). These include most profoundly regions of the anterior cingulate that are completely (*R* = 1.0) hyperconnected to each other and not to any other ROI. ROIs in and around the right frontal lobe (11, 10, 46, 47) also seem to form a loop of highly connected activity while their connections to other regions are quite limited. The fusiform gyrus is highly connected to the posterior cingulate and pre-cuneus, but again not to other ROIs. What is missing is a link between the fusiform gyrus, superior temporal gyrus, insula and inferior frontal regions that forms the social perceptual system (Pelphrey et al., 2004). This important neuronal system appears to be underconnected in this case.

**Table 2:** **Results for the sLORETA correlation analyses**.

		**Correlations**														
		**BA45**	**BA47**	**BA46**	**BA10**	**BA11**	**BA13**	**BA30**	**BA20**	**BA23**	**BA33**	**BA28**	**BA31**	**BA24**	**BA37**	**BA38**
BA45	Pearson correlation	1	0.584	0.940	0.381	0.358	0.977^*^	0.553	0.922	0.782	0.547	0.712	0.927	0.531	0.802	0.607
	Sig. (2-tailed)		0.416	0.060	0.619	0.642	0.023	0.447	0.078	0.218	0.453	0.288	0.073	0.469	0.198	0.393
	*N*	4	4	4	4	4	4	4	4	4	4	4	4	4	4	4
BA47	Pearson correlation	0.584	1	0.817	0.968^*^	0.967^*^	0.413	0.909	0.728	0.910	0.883	0.976^*^	0.804	0.889	0.739	0.993^**^
	Sig. (2-tailed)	0.416		0.183	0.032	0.033	0.587	0.091	0.272	0.090	0.117	0.024	0.196	0.111	0.261	0.007
	*N*	4	4	4	4	4	4	4	4	4	4	4	4	4	4	4
BA46	Pearson correlation	0.940	0.817	1	0.670	0.644	0.848	0.723	0.918	0.896	0.783	0.886	0.962^*^	0.773	0.823	0.820
	Sig. (2-tailed)	0.060	0.183		0.330	0.356	0.152	0.277	0.082	0.104	0.217	0.114	0.038	0.227	0.177	0.180
	*N*	4	4	4	4	4	4	4	4	4	4	4	4	4	4	4
BA10	Pearson correlation	0.381	0.968^*^	0.670	1	0.994^**^	0.185	0.829	0.533	0.781	0.896	0.891	0.630	0.906	0.559	0.941
	Sig. (2-tailed)	0.619	0.032	0.330		0.006	0.815	0.171	0.467	0.219	0.104	0.109	0.370	0.094	0.441	0.059
	*N*	4	4	4	4	4	4	4	4	4	4	4	4	4	4	4
BA11	Pearson correlation	0.358	0.967^*^	0.644	0.994^**^	1	0.167	0.871	0.546	0.800	0.845	0.898	0.633	0.858	0.597	0.951^*^
	Sig. (2-tailed)	0.642	0.033	0.356	0.006		0.833	0.129	0.454	0.200	0.155	0.102	0.367	0.142	0.403	0.049
	*N*	4	4	4	4	4	4	4	4	4	4	4	4	4	4	4
BA13	Pearson correlation	0.977^*^	0.413	0.848	0.185	0.167	1	0.434	0.882	0.678	0.361	0.572	0.858	0.342	0.760	0.450
	Sig. (2-tailed)	0.023	0.587	0.152	0.815	0.833		0.566	0.118	0.322	0.639	0.428	0.142	0.658	0.240	0.550
	*N*	4	4	4	4	4	4	4	4	4	4	4	4	4	4	4
BA30	Pearson correlation	0.553	0.909	0.723	0.829	0.871	0.434	1	0.806	0.951^*^	0.609	0.946	0.826	0.619	0.890	0.952^*^
	Sig. (2-tailed)	0.447	0.091	0.277	0.171	0.129	0.566		0.194	0.049	0.391	0.054	0.174	0.381	0.110	0.048
	*N*	4	4	4	4	4	4	4	4	4	4	4	4	4	4	4
BA20	Pearson correlation	0.922	0.728	0.918	0.533	0.546	0.882	0.806	1	0.937	0.522	0.858	0.989^*^	0.515	0.970^*^	0.779
	Sig. (2-tailed)	0.078	0.272	0.082	0.467	0.454	0.118	0.194		0.063	0.478	0.142	0.011	0.485	0.030	0.221
	*N*	4	4	4	4	4	4	4	4	4	4	4	4	4	4	4
BA23	Pearson correlation	0.782	0.910	0.896	0.781	0.800	0.678	0.951^*^	0.937	1	0.685	0.978^*^	0.958^*^	0.686	0.951^*^	0.946
	Sig. (2-tailed)	0.218	0.090	0.104	0.219	0.200	0.322	0.049	0.063		0.315	0.022	0.042	0.314	0.049	0.054
	*N*	4	4	4	4	4	4	4	4	4	4	4	4	4	4	4
BA33	Pearson correlation	0.547	0.883	0.783	0.896	0.845	0.361	0.609	0.522	0.685	1	0.807	0.642	1000^**^	0.439	0.824
	Sig. (2-tailed)	0.453	0.117	0.217	0.104	0.155	0.639	0.391	0.478	0.315		0.193	0.358	0.000	0.561	0.176
	*N*	4	4	4	4	4	4	4	4	4	4	4	4	4	4	4
BA28	Pearson correlation	0.712	0.976^*^	0.886	0.891	0.898	0.572	0.946	0.858	0.978^*^	0.807	1	0.908	0.810	0.866	0.990^*^
	Sig. (2-tailed)	0.288	0.024	0.114	0.109	0.102	0.428	0.054	0.142	0.022	0.193		0.092	0.190	0.134	0.010
	*N*	4	4	4	4	4	4	4	4	4	4	4	4	4	4	4
BA31	Pearson correlation	0.927	0.804	0.962^*^	0.630	0.633	0.858	0.826	0.989^*^	0.958^*^	0.642	0.908	1	0.635	0.946	0.839
	Sig. (2-tailed)	0.073	0.196	0.038	0.370	0.367	0.142	0.174	0.011	0.042	0.358	0.092		0.365	0.054	0.161
	*N*	4	4	4	4	4	4	4	4	4	4	4	4	4	4	4
BA24	Pearson correlation	0.531	0.889	0.773	0.906	0.858	0.342	0.619	0.515	0.686	1000^**^	0.810	0.635	1	0.437	0.830
	Sig. (2-tailed)	0.469	0.111	0.227	0.094	0.142	0.658	0.381	0.485	0.314	0.000	0.190	0.365		0.563	0.170
	*N*	4	4	4	4	4	4	4	4	4	4	4	4	4	4	4
BA37	Pearson correlation	0.802	0.739	0.823	0.559	0.597	0.760	0.890	0.970^*^	0.951^*^	0.439	0.866	0.946	0.437	1	0.807
	Sig. (2-tailed)	0.198	0.261	0.177	0.441	0.403	0.240	0.110	0.030	0.049	0.561	0.134	0.054	0.563		0.193
	*N*	4	4	4	4	4	4	4	4	4	4	4	4	4	4	4
BA38	Pearson correlation	0.607	0.993^**^	0.820	0.941	0.951^*^	0.450	0.952^*^	0.779	0.946	0.824	0.990^*^	0.839	0.830	0.807	1
	Sig. (2-tailed)	0.393	0.007	0.180	0.059	0.049	0.550	0.048	0.221	0.054	0.176	0.010	0.161	0.170	0.193	
	*N*	4	4	4	4	4	4	4	4	4	4	4	4	4	4	4

* Correlation is significant at the 0.05 level (2-tailed).

** Correlation is significant at the 0.01 level (2-tailed).

**Table 3:** **SIFT/GCC maximal values between ICs**.

**From To**	**1**	**2**	**3**	**5**	**8**	**9**	**10**	**15**	**18**	**19**
1		0.57	0.50	0.59	0.21	0.22	0.89	0.14	0.36	0.12
2	0.36		0.49	1.51	0.26	0.10	0.50	0.15	0.11	0.28
3	0.04	1.09		0.15	0.52	0.1	0.28	0.10	0.24	0.80
5	0.85	1.31	0.39		0.13	0.09	0.34	0.31	0.05	0.84
8	0.61	0.51	0.82	0.2		0.38	0.99	0.29	1.04	0.13
9	0.24	0.29	0.24	0.08	0.28		0.48	1.22	0.29	0.17
10	1.35	0.35	0.46	0.19	0.72	0.19		0.38	0.92	0.48
15	0.30	0.26	0.41	0.11	0.34	1.07	0.87		0.30	0.15
18	0.39	0.08	0.74	0.11	1.18	0.26	1.87	0.17		0.29
19	0.40	0.66	2.08	2.39	0.31	0.19	1.32	0.18	0.72	

Independent components included: 1 (Brodmann area (BA) 32; Anterior Cingulate), 2 (BA 10; Middle Frontal Gyrus), 3 (BA 40; Inferior Parietal Lobule), 5 (BA 10; Middle Frontal Gyrus), 8 (BA 37; Fusiform Gyrus), 9 (BA 19; Lingual Gyrus), 10 (BA 40; Inferior Parietal Lobule), 15 (BA 22; Superior Temporal Gyrus), 18 (BA 18; Cuneus), and 19 (BA 10; Middle Frontal Gyrus).

## Effective connectivity as measured by granger causality

One of the critiques of other coherence methods has been that they are largely based on the concept of correlation or similarity. Even sLORETA coherence is still the similarity between sources of EEG activity. An advanced statistical technique for investigated directed causation that uses multiple autoregressive analyses is Granger causality and it's related concepts of partial directed coherences (Seth, 2010). Granger causality analysis (GCA) is a method for investigating whether one time series can correctly forecast another (Bressler and Seth, 2010). Granger causality (GC) is a data-driven approach based on linear regressive models and requires only a few basic assumptions about the original data statistics. Recently in neuroscience applications, GC has been used to explore causal dependencies between brain regions by investigating directed information flow or causality in the brain. It uses the error prediction of autoregressive (AR) or multi-variant autoregressive (MAR) models to estimate if a brain process is a Granger-cause of another brain process.

### Methods

To perform such an analysis on this same EEG data stream as used in the two examples above, we utilized the SIFT (Source Information Flow Toolbox) toolbox from EEGLAB v.12 (Delorme et al., 2011). A key aspect of SIFT is that it focuses on estimating and visualizing multivariate effective connectivity in the source domain rather than between scalp electrode signals. This should allow us to achieve finer spatial localization of the network components while minimizing the challenging signal processing confounds produced by broad volume conduction from “neural” sources to the scalp electrodes. From our eyes open resting EEG data we have virtually epoched this stream into 1-s segments. Independent Component Analysis was then used to extract unique, independent components from the data. To fit multiple component dipoles and determine their locations DIPFIT toolbox was then applied. Then by investigating the dipole locations and the components topographical maps, only good “neural” components that are related to neural process in the brain have been included for further processing. These data were then fit into a MAR model using Vieira-Morf algorithm. For our data the model and after some trials and errors and model validation process, the MAR model order has been set to 5. In addition, the frequency band of interest has been selected from 1 to 30 Hz and the most obvious connectivity measure was Grager-Geweke Causality (GGC).

**Figure 4:**
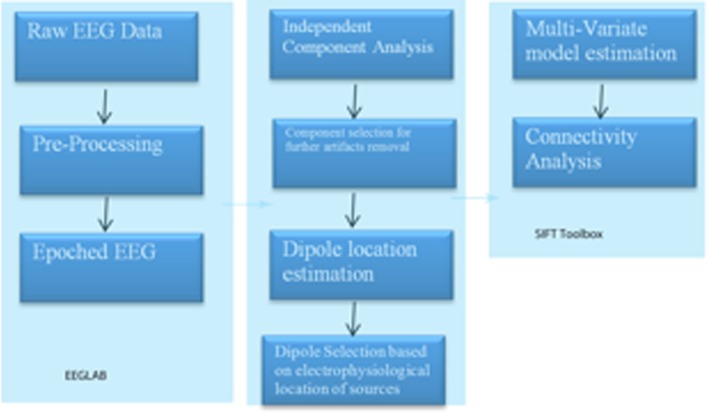
**SIFT/Granger (GGC) causality sequence of processing**.

These methods of operation are summarized in Figure 4. This takes the EEG data from sensory to source space via independent component analysis and dipole localization. This diminishes the issue of volume conduction (see Astolfi et al., 2007; Akalin Acar and Makeig, 2013). Once dipole localization has been performed, these data are subjected to MVAR and Granger Causality (GC) analysis as presented above. Within a reasonable range of values, changes in model order may show little effect on the spectral density (and by extension coherence) (e.g., see Florian and Pfurtscheller, 1995). Our model order has been based on Akaike Information Criterion (AIC) and Bayesian Information Criterion (BIC) criteria to maximize model effects. Statistically, the critical issue for GC is the ratio between the number of independent observations (i.e., samples) and the model complexity (i.e., number of parameters). If the number of observations is large relative to the number of parameters then the model order selection criteria are still valid. If the number of observations is small, then we might run into problems with AIC and other asymptotic estimators, but there are corrections for that (corrected akaike information criterion). In our data set (case epoching), we have plenty of data available and the ratio of observations [total data samples within a time window (x trials)] to parameters is >40 suggesting that we have a valid model using AIC (Burnham, 2004).

### Results

**Figure 5:**
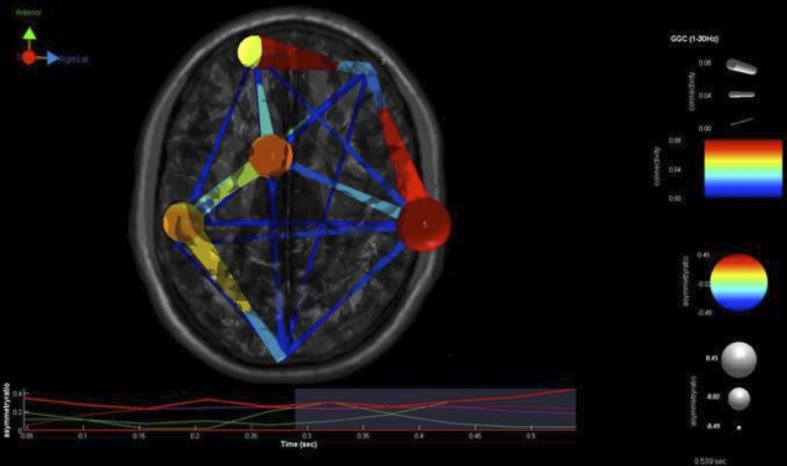
**SIFT/Granger (GGC) causality brain image**. Levels of greater connectivity are shown with thicker lines and brighter colors. Direction of causality is indicated by the key in the upper left hand corner. ICs and their localization are listed as part of Table 3.

Our findings for this case are presented in Figure 5. This, again demonstrates regions of over and under-connectivity. There appear to be several regions of heightened causality whose major influence is only toward close neighbors. This includes regions of the prefrontal cortex, anterior cingulate, and bilateral inferior parietal lobules. In each instance, these regions are somewhat isolated from each other and other important ICs as well. What is also clear is that there are long connections throughout the right hemisphere that are largely under-connected. These span as far away as the cuneus to the inferior frontal gyrus and include regions of the temporal lobes and underlying areas such as the fusiform gyrus and superior temporal gyrus.

## Comparison of coherence techniques

While it has not been shown, a pairwise coherence analysis of this case has shown very few significant coherence anomalies. The ones that are present include frontal hypocoherence and bilateral occipital-temporal hypocoherences. This is the opposite of what is shown in the multivariate analyses. All forms of multivariate analysis shown have suggested a combination of local hypercoherence and long distance hypocoherence across right frontal to posterior temporolimbic regions. This, in this case, clearly shows a difference between pairwise and multivariate estimates.

Comparing these to know structural connectivity was possible in this case in the form of MR-DTI analysis within this same system of concern (mirror neuron system). This suggests the presence of prefrontal and anterior cingulate hyperconnectivity and dramatic hypoconnectivity from frontal to temporolimbic regions. Comparing this to the multivariate analyses is interesting as there is similarity across all of these. The resemblance of these measures of functional connectivity to the reality of structural connectivity in this case is seen in its' greatest detail in multivariate measures that localize to source space (sLoreta, SIFT GC). As such, one limitation of the first method (Hudspeth NREP) is that it does not source localize activit prior to generating eigenimages of sensory covariances. GC has certain possible advantages including measuring the degree, directionality of connectivity, reciprocal influences and localization to regions that are deeper than is possible with sLoreta. It should be recalled that these observations are based on theory and one a single case study. Clearly, much more research is needed in this area of study.

## Discussion

Neuroimaging technologies and research has shown that autism is largely a disorder of neuronal connectivity. While advanced work is being done with fMRI, MRI-DTI, SPECT and other forms of structural and functional connectivity analyses, the use of EEG for these purposes is of additional great utility. Cantor et al. (1986) were the first to examine the utility of pairwise coherence measures for depicting connectivity impairments in autism. Since that time research has shown a combination of mixed over and under-connectivity that is at the heart of the primary symptoms of this multifaceted disorder. Nevertheless, there is reason to believe that these simplistic pairwise measurements under represent the true and quite complicated picture of connectivity anomalies in these persons. We have presented three different forms of multivariate connectivity analysis with increasing levels of sophistication. These all seem able to capture the complexity of such cases and certainly moreso than pairwise estimates have. There does appear to be a value in using measures that localize the source of EEG activity and judge coherence from these sources. Further, the promise of using MVAR advanced statistical methods to judge effective connectivity and causation is exciting.

Clearly, there is much work to be done to further the scientific underpinnings of these approaches. Future work should extend these forms of analysis to greater sample sizes of autistic children and adults to judge their validity and utility. Comparing findings from autistics to other diagnostic and typically developing samples will be crucial. Lastly, the true value of any form of assessment for autistic children may be in it's applicability to further treatment outcomes for these children. Coben (2013) has shown that such metrics may be used to engineer more effective treatment plans than traditional neurofeedback with impressive outcomes as a result. It is hoped that advancements with such assessment techniques will further sharpen such treatment successes and decrease durations of treatment.

### Conflict of interest statement

The authors declare that the research was conducted in the absence of any commercial or financial relationships that could be construed as a potential conflict of interest.
